# Skeletal muscle regeneration in facioscapulohumeral muscular dystrophy is correlated with pathological severity

**DOI:** 10.1093/hmg/ddaa164

**Published:** 2020-08-03

**Authors:** Christopher R S Banerji, Don Henderson, Rabi N Tawil, Peter S Zammit

**Affiliations:** Randall Centre for Cell and Molecular Biophysics, King’s College London, London SE1 1UL, UK; Neuromuscular Pathology Laboratory, Department of Neurology, University of Rochester Medical Center, Rochester, NY 14642, USA; Neuromuscular Unit, Department of Neurology, University of Rochester Medical Center, Rochester, NY 14642, USA; Randall Centre for Cell and Molecular Biophysics, King’s College London, London SE1 1UL, UK

## Abstract

Facioscapulohumeral muscular dystrophy (FSHD) is an autosomal-dominant myopathy characterized by slowly progressive skeletal muscle weakness and wasting. While a regenerative response is often provoked in many muscular dystrophies, little is known about whether a regenerative response is regularly elicited in FSHD muscle, prompting this study. For comparison, we also examined the similarly slowly progressing myotonic dystrophy type 2 (DM2). To first investigate regeneration at the transcriptomic level, we used the 200 human gene Hallmark Myogenesis list. This myogenesis biomarker was elevated in FSHD and control healthy myotubes compared to their myoblast counterparts, so is higher in myogenic differentiation. The myogenesis biomarker was also elevated in muscle biopsies from most independent FSHD, DM2 or Duchenne muscular dystrophy (DMD) studies compared to control biopsies, and on meta-analysis for each condition. In addition, the myogenesis biomarker was a robust binary discriminator of FSHD, DM2 and DMD from controls. We also analysed muscle regeneration at the protein level by immunolabelling muscle biopsies for developmental myosin heavy chain. Such immunolabelling revealed one or more regenerating myofibres in 76% of FSHD muscle biopsies from quadriceps and 91% from tibialis anterior. The mean proportion of regenerating myofibres per quadriceps biopsy was 0.48%, significantly less than 1.72% in the tibialis anterior. All DM2 muscle biopsies contained regenerating myofibres, with a mean of 1.24% per biopsy. Muscle regeneration in FSHD was correlated with the pathological hallmarks of fibre size variation, central nucleation, fibrosis and necrosis/regeneration/inflammation. In summary, the regenerative response in FSHD muscle biopsies correlates with the severity of pathology.

## Introduction

Facioscapulohumeral muscular dystrophy (FSHD) is characterized by slowly progressive skeletal muscle weakness and wasting, starting with facial muscles and progressing to the shoulder girdle, proximal upper limb and lower limb muscles (1,2). FSHD has a prevalence of 1 in 8300 (3).

Genetic diagnosis divides FSHD into FSHD1 (OMIM: 158900) that accounts for ~95% of cases, with the remainder classified as FSHD2 (OMIM: 158901). However, clinical diagnosis does not distinguish between the two conditions, indicating a common pathomechanism (2). FSHD1 is associated with partial deletion in the D4Z4 macrosatellite repeat array in the subtelomere of chromosome 4 at 4q35. FSHD1 is characterized by the presence of 1–10 D4Z4 repeats on at least one 4q chromosome, while the unaffected population normally have between 11 and ~100 D4Z4 units (4–6). So few D4Z4 units in FSHD1 patients leads to epigenetic derepression at the locus, including DNA hypomethylation and chromatin relaxation (7,8). Crucially, at least one D4Z4 unit is required for FSHD (9). FSHD2 does not usually display such an extensive reduction in the number of D4Z4 units (typically 12–16 on at least one chromosome 4), but epigenetic changes occur due to mutations in proteins needed for maintaining epigenetic repression at D4Z4, principally Structural Maintenance of Chromosomes Flexible Hinge Domain Containing 1 (SMCHD1) (OMIM: 614982) (10). *SMCHD1* mutations can also act as a disease modifier in FSHD1 (11).

Each D4Z4 repeat contains a retrogene encoding for a double homeobox transcription factor termed *Double homeobox 4* (*DUX4*) (OMIM: 606009) (12,13). Restricted to old world primates, DUX4 is a pioneer transcription factor (14) that drives transcription of genes and retroelements during the cleavage stage of early development to control zygotic genome activation (15,16). DUX4 is then repressed in somatic cells via epigenetic modification at D4Z4 (7,8). In FSHD, however, epigenetic derepression at D4Z4 causes transcription of *DUX4* from the normally somatically repressed distal-most D4Z4 unit (17). Importantly, in addition to epigenetic derepression at D4Z4, FSHD also requires a polymorphism in *cis* in the flanking telomeric pLAM region that provides a polyadenylation signal in a non-coding exon 3 of the *DUX4* transcript in particular 4qA haplotypes (e.g. 4qA161 and rarer 4qA159 and 4qA168) (18). Thus, divergent genomic alterations for FSHD1 and FSHD2 manifest as a coherent clinical condition through D4Z4 epigenetic derepression on a 4qA chromosome that permits *DUX4* expression from the distal-most D4Z4 unit, whose transcripts are then stabilized by splicing to a downstream poly(A) signal-containing exon on permissive 4qA haplotypes (7,10).

Muscular dystrophies are hallmarked by skeletal muscle weakness and wasting, and muscle regeneration in response to damage is evident in many such disorders, such as Duchenne muscular dystrophy (DMD) (19,20). Skeletal muscle has a remarkable capacity to regenerate in response to most insult, due to a population of resident stem cells called satellite cells (21,22). In response to damage, satellite cells activate and proliferate to provide myoblasts that either self-renew, or differentiate to replace lost myonuclei: redeploying many regulatory factors/pathways that control developmental myogenesis, including *Pax* genes (21–25). However, progressive muscle wasting shows that regeneration does not prevent dystrophic changes. This is in part likely because satellite cells are operating in an increasing hostile microenvironment displaying chronic inflammation and increasing fibrosis (26). In addition, in some muscular dystrophies, the pathogenic mutation that elicits muscle fibre damage may also affect satellite cell function to further compromise any regenerative outcome (27,28).

Satellite cells are present in FSHD muscle biopsies in similar numbers to controls (29). However, evidence of muscle regeneration in FSHD is scant. Padberg (1) reported regenerating muscle fibres in <30% of muscle biopsies taken from several different muscles using histological criteria, in the era before genetic diagnosis. Similarly, muscle biopsies from patients with a clinical presentation of FSHD had small angular fibres, immature 2C fibres and alkaline phosphatase activity, associated with denervated or regenerating muscle fibres (30). Although directed at assessing the inflammatory response, Arahata et al. (31) also noted regenerating myofibres based on histological criteria in 67% of biceps brachii biopsies, but again without genetic diagnosis for the majority of patients. Histology alone, though, is limited for sensitively and definitively identifying regenerating muscle fibres. Better is immunolabelling for developmental isoforms of certain proteins such as myosin heavy chain (MyHC) (32), and fetal MyHC has been illustrated in FSHD muscle biopsies (33), but without quantification or indication of how representative such observations are.

To address the central question of how common is muscle regenerative in FSHD, we performed a large-scale systematic investigation at the transcriptomic and protein levels. We first examined regeneration at the transcriptomic level by assaying the mean expression of 200 human genes comprising the HALLMARK_MYOGENESIS gene set (34). This myogenesis biomarker was elevated in most independent FSHD muscle biopsies and on meta-analysis compared to controls and was also a good discriminator of FSHD versus control biopsies. For comparison, we also examined the similarly slowly progressing myotonic dystrophy type 2 (DM2), caused by expansion of a tetranucleotide repeat (CCTG*CAGG)n in intron 1 of *CNBP* (*ZNF9*) and in overlapping antisense genes (35). Again, the myogenesis biomarker was significantly elevated in DM2 muscle biopsies compared to controls and was a very good discriminator of DM2 versus control biopsies, as it also was for DMD. To next determine the frequency and extent of muscle regeneration, we immunolabelled muscle biopsies for developmental MyHC. Regenerating myofibres were evident in 76% (26/34 FSHD patients) of muscle biopsies from the quadriceps, with a mean 0.48% regenerating myofibres per biopsy. For the tibialis anterior, 91% (10/11 FSHD patients) of biopsies contained regenerating muscle fibres, averaging 1.72% regenerating myofibres per biopsy. For DM2, all muscle biopsies (9/9 DM2 patients) contained regenerating muscle fibres with a mean proportion of 1.24% per biopsy. Pathological evaluation of the biopsies (36) revealed a correlation between the proportion of regenerating muscle fibres and the severity of pathology in FSHD.

## Results

### The HALLMARK_MYOGENESIS biomarker validates on myogenic differentiation in human myoblasts

Gene set enrichment analysis on our transcriptomic dataset describing myogenic differentiation in immortalized healthy and FSHD1 myoblasts at eight time points (37) identified HALLMARK_MYOGENESIS as the gene set most significantly upregulated during myogenic differentiation, regardless of disease status. The HALLMARK_MYOGENESIS gene set consists of 200 human genes associated with myogenesis (Supplementary Material, Table S1), which was generated from the Molecular Signatures Database by integration and refinement of 64 independent gene expression studies investigating myogenesis (34). This core set of 200 genes was then cross-validated to ensure association with myogenic progression rather than other cellular differentiation processes, revealing the gene set as specific to myogenesis (34). Our hypothesis was that the average expression of HALLMARK_MYOGENESIS genes (hereafter called the Myogenesis score) in any given adult muscle sample detects muscle regeneration.

**Figure 1:**
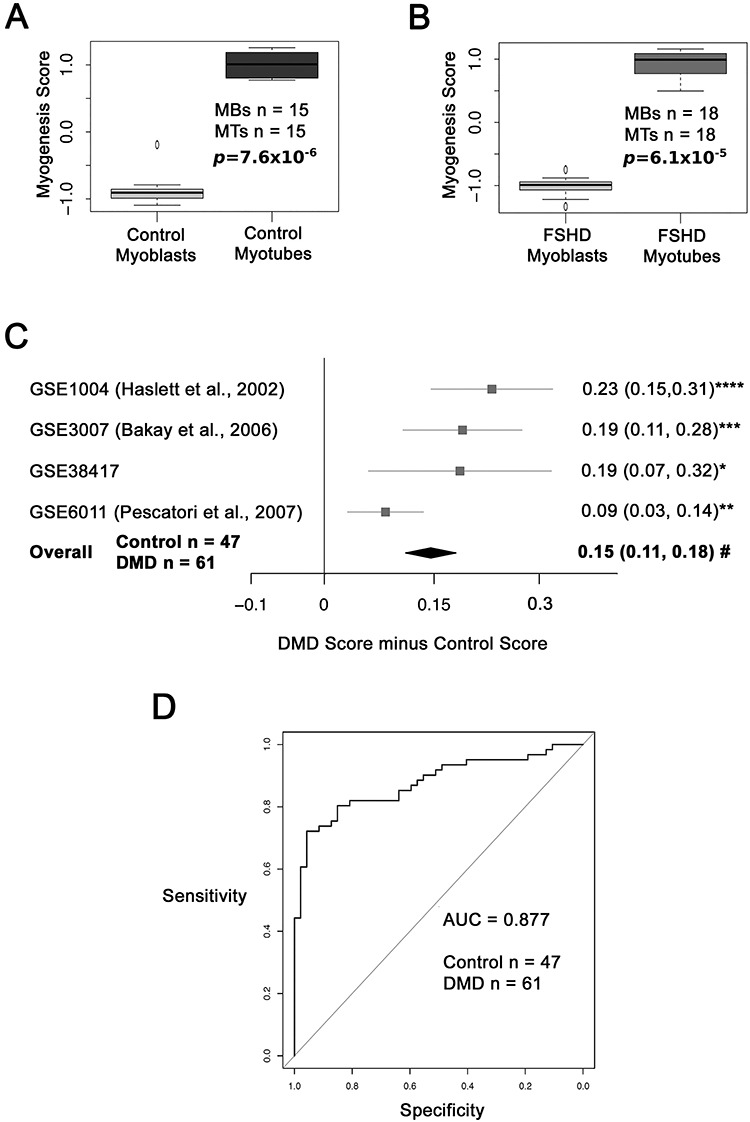
The Myogenesis score is higher in DMD. (**A**) A box plot shows that the Myogenesis score is higher in RNA-seq data from healthy control myotubes than from healthy control myoblasts. (**B**) Box plot showing that the Myogenesis score is also elevated in transcriptomic data from FSHD1 myotubes compared to FSHD1 myoblasts. The box represents the interquartile range (IQR), with the median indicated by a line. Whiskers denote min [1.5*IQR, max (observed value)]. ‘o’ represents data points greater than 1.5 IQR from the median, *n* = 15 control, and *n* = 18 FSHD, myoblasts (MBs) and myotube (MTs) datasets. *P* values are given. (**C**) A forest plot shows that the Myogenesis score is elevated across all four published DMD muscle biopsy data sets and on meta-analysis (*n* = 61 DMD and *n* = 47 control muscle biopsies). The differential scores (DMD score minus control score) alongside 95% confidence intervals are provided. For single studies, a two-tailed Wilcoxon *U*-test was performed to assess significance, where an asterisk denotes *P* < 0.05, two asterisks denote *P* < 0.005, three asterisks denote *P* < 0.0005 and four asterisks denote *P* < 0.00005. A Fisher’s combined test was employed for overall assessment where hash indicates *P* = 1.5 × 10^−10^. (**D**) A ROC curve compares the discriminatory power of the Myogenesis score across the four DMD muscle biopsy datasets pooled. The AUC is 0.877, demonstrating that the Myogenesis score is a robust discriminator of DMD status.

To first validate the Myogenesis score, we computed it on our RNA-seq dataset of immortalized and primary human myoblasts from healthy and FSHD1 patients (37–39). Myoblasts were profiled by RNA-seq both in proliferation medium at confluency and on day 3.5 of myogenic differentiation, when large myotubes were present. The Myogenesis score was significantly elevated in healthy myotubes compared to healthy myoblasts, confirming that a high Myogenesis score associates with myogenic differentiation (Fig. 1A). The Myogenesis score was also significantly elevated in FSHD1 myotubes compared to FSHD1 myoblasts (Fig. 1B).

### The Myogenesis score is higher in muscle biopsies from DMD patients

A robust regenerative response to pathology is observed initially in both DMD and its mouse models, as shown by the presence of many muscle fibres expressing developmental isoforms of MyHC (Dev MyHC) (19,20,40). To validate the Myogenesis score *in vivo*, we analysed four transcriptomic datasets comprising 61 DMD and 47 control healthy muscle biopsies (41–43). The Myogenesis score was significantly higher in each individual DMD dataset versus control and on meta-analysis (Fisher’s combined *P* = 1.5 × 10^−10^) (Fig. 1C).

Quantification of the Myogenesis score was also performed using receiver operating characteristic (ROC) curve analysis, which depicts performance of a binary classifier at different threshold values. Area under the curve (AUC) represents the probability that the Myogenesis score will on average discriminate a DMD sample from a control. ROC curve analysis on the pooled four DMD datasets demonstrated an AUC of 0.877 for the Myogenesis score as a DMD biomarker (Fig. 1D), making it a robust discriminator of DMD muscle biopsies from control.

### The Myogenesis score is elevated in FSHD patient muscle biopsies

**Figure 2:**
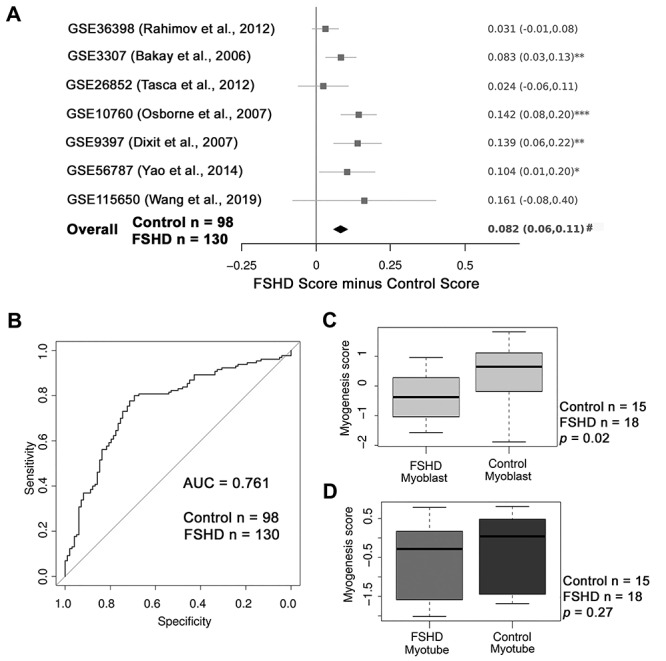
The Myogenesis score is elevated in FSHD muscle biopsies. (**A**) A forest plot shows that the Myogenesis score is elevated in FSHD muscle biopsies compared to relevant controls in four of seven independent published studies and on meta-analysis across the seven FSHD muscle biopsy data sets (*n* = 130 FSHD and *n* = 98 control muscle biopsies). The differential scores (FSHD score minus control score) alongside 95% confidence intervals are provided. For single studies, a two-tailed Wilcoxon *U*-test was performed to assess significance, where asterisks denote *P* < 0.05. A Fisher’s combined test was employed for overall assessment, where hash indicates *P* = 3.23 × 10^−7^. (**B**) A ROC curve compares the discriminatory power of the Myogenesis score across the pooled seven FSHD muscle biopsy datasets. The AUC is 0.761, demonstrating that the Myogenesis score is a good discriminator of FSHD status. (**C**) Box plot illustrates that the Myogenesis score is significantly lower in RNA-seq data from FSHD myoblasts compared to control myoblasts. (**D**) Box plot shows that the Myogenesis score is not significantly different between FSHD and control myotubes. Boxes represent the IQR, with the median indicated by a line. Whiskers denote min [1.5*IQR, max (observed value)], *n* = 15 control and *n* = 18 FSHD, with the *P* values given.

We next used the Myogenesis score to examine gene expression in seven independent published FSHD muscle biopsy datasets, profiling 130 FSHD and 98 control healthy samples (17,42,44–48). Meta-analysis revealed that the FSHD samples had a significantly higher Myogenesis score than controls as assessed using Fisher’s combined probability test (*P* = 3.23 × 10^−7^) and attained significance on four of seven independent datasets (Fig. 2A).

**Figure 3:**
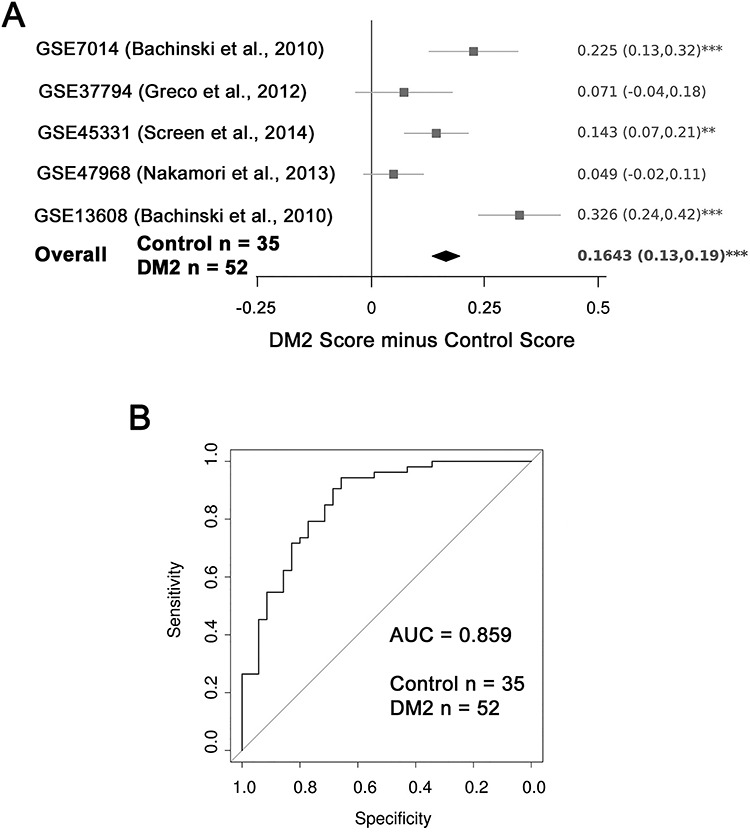
DM2 muscle biopsies have a higher Myogenesis score than controls. (**A**) A forest plot reveals that the Myogenesis score is elevated in three of five published DM2 muscle biopsy data sets and on meta-analysis (*n* = 52 DM2 and *n* = 35 control muscle biopsies). The differential scores (DM2 score minus control score) alongside 95% confidence intervals are provided. For single studies, a two-tailed Wilcoxon *U*-test was performed to assess significance, while a Fisher’s combined test was employed for overall assessment: asterisks denote *P* < 0.05. (**B**) A ROC curve compares the discriminatory power of the Myogenesis score across the pooled five DM2 muscle biopsy datasets. The AUC is 0.859, demonstrating that the Myogenesis score is a very good discriminator of DM2 status.

ROC curve analysis on the pooled seven FSHD datasets demonstrated an AUC of 0.761 for the Myogenesis score as an FSHD biomarker (Fig. 2B), making it a good discriminator of FSHD muscle biopsies from control.

We also computed the Myogenesis score on FSHD versus control (healthy) myoblasts and FSHD versus control (healthy) myotubes. FSHD myoblasts had a significantly lower Myogenesis score than controls (Fig. 2C), while there was no difference in the Myogenesis score between FSHD and control myotubes (Fig. 2D).

### Myotonic dystrophy type 2 has an elevated Myogenesis score

We also examined DM2 to determine if a similarly slowly progressing muscular dystrophy (35) also has a higher Myogenesis score than healthy controls*.* Five published DM2 transcriptomic data sets were used, profiling 52 DM2 muscle biopsies alongside 35 controls (49–52). The Myogenesis score was significantly elevated in DM2 muscle biopsies compared to controls in three of five independent studies and on meta-analysis (Fig. 3A).

ROC curve analysis on the five datasets pooled demonstrated an AUC of 0.859 for the Myogenesis score as a DM2 biomarker (Fig. 3B), making it a robust discriminator of DM2 muscle biopsies from healthy controls.

### Regenerating muscle fibres detected in FSHD muscle biopsies

Having established increased expression of genes associated with myogenesis in both FSHD and DM2 muscle biopsies, we next examined muscle biopsies for regenerating muscle fibres at the protein level using immunolabelling. Our cohort of 45 FSHD patients consisted of both FSHD1 (*n* = 41) and FSHD2 (*n* = 4), with a mean age of 50 years (range: 24–75 years) and was 49% female. Needle muscle biopsies were obtained from quadriceps muscles in 34 patients (Table 1) and tibialis anterior in 11 subjects (Table 2). The genetically confirmed DM2 study cohort included nine subjects (56% female), with a mean subject age of 58 years (range: 44–65 years). Needle muscle biopsies were obtained from tibialis anterior in five DM2 subjects or quadriceps in four subjects (Table 3).

Separate cryosections of the muscle biopsies were stained with haematoxylin and eosin (H&E) and trichrome and evaluated using a 0–12 point Pathology Score totalled from four categories each scored 0–3 comprising fibre size variation, central nucleation, fibrosis and the combined presence of necrosis and/or regeneration and/or inflammation (36). Pathologic scores of 1–4 are classified as mild, 5–8 moderate and 9–12 severe (36). The mean ± standard error of the mean (SEM) pathology score for the FSHD quadriceps biopsies was 3.94 ± 0.44 (*n* = 34), at the top end of the mild (1–4) category (Table 1). For the FSHD tibialis anterior biopsies, the mean ± SEM pathology score was 5.27 ± 0.97 (*n* = 11), within the moderate (5–8) category (Table 2). For DM2, the mean ± SEM pathology score was 6.78 ± 0.64 (*n* = 9), so of moderate severity (Table 3).

Dev MyHC isoforms are transiently expressed in regenerating muscle fibres, before being replaced by adult isoforms as the myofibre matures (32). To detect regenerating muscle fibres, muscle biopsy cryosections were immunolabelled for Dev MyHC using Novocastra NCL-MHCd (Clone RNMy2/9D2) that recognizes MyHC expressed during skeletal muscle development. Histological staining of some muscle biopsies for non-specific esterase revealed little, if any, overlap with Dev MyHC immunolabelling, confirming myofibres as regenerating, rather than denervated, muscle fibres (data not shown).

**Figure 4:**
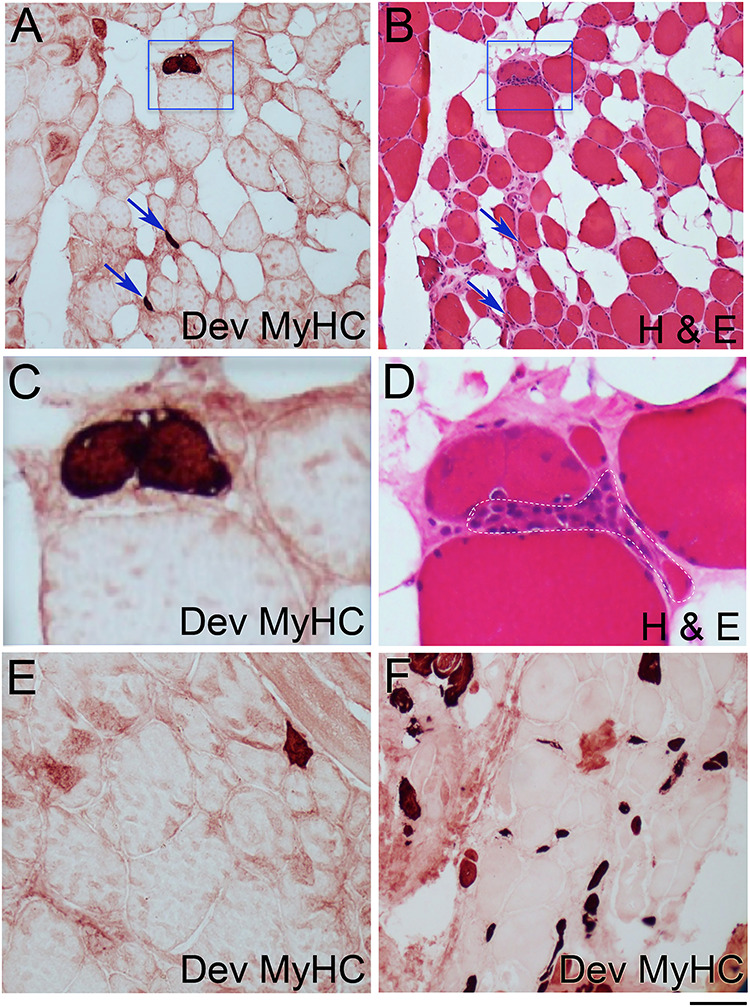
Regenerating muscle fibres are present in most FSHD muscle biopsies. (**A**) Histopathological section from a quadriceps biopsy from a FSHD patient immunolabelled for Novocastra NCL-MHCd (Clone RNMy2/9D2). Regenerating myofibres containing Dev MyHC are highlighted by arrows or a blue box. (**B**) Adjacent section to that in (A) stained with H&E with the same Dev MyHC-containing regenerating muscle fibres again highlighted. (**C**) Area delimited by the blue box in (A) at higher magnification to better show the two Dev MyHC-containing regenerating muscle fibres. (**D**) Area delimited by blue box from (B) shows that the Dev MyHC-containing muscle fibres have histological feature associated with regeneration, including more basophilic stippling in the cytoplasm due to the increased RNA content and big, plump, less dense myonuclei, associated with a more active nucleus. Area delimited by white dashed line encompasses a necrotic muscle fibre undergoing phagocytosis, as the cytoplasm appears fragmented, without the usual uniform pink eosinophilic staining. (**E**) Dev MyHC-containing regenerating muscle fibre in an area of muscle exhibiting less overt signs of pathology. (**F**) Tibialis anterior section showing many Dev MyHC-containing regenerating muscle fibres. Scale bar represents approximately 125 μm (A, B and F), 30 μm (C and D) or 50 μm (E).

For FSHD, 76.5% (26/34) of muscle biopsies from quadriceps (Fig. 4A–E) and 90.9% (10/11) from tibialis anterior (Fig. 4F) contained one or more muscle fibres containing Dev MyHC, indicating active muscle regeneration (Tables 1 and 2). In contrast, no myofibres contained Dev MyHC in control muscle biopsies taken from two healthy adults (data not shown). There was a mean ± SEM of 0.48 ± 0.15% regenerating muscle fibres per quadriceps biopsy (*n* = 34), significantly less than 1.72 ± 0.86% (*n* = 11) in the tibialis anterior (*t*-value: 2.28, *P* = 0.027 using logistic regression) (Tables 1 and 2). The tibialis anterior tends to be affected earlier than the quadriceps in FSHD progression, which was reflected in an overall classification of moderate severity pathology for the tibialis anterior compared to an average mild categorization for the quadriceps (Tables 1 and 2).

FSHD muscle biopsies classified as severe generally contained higher proportions of regenerating muscle fibres. Considering just the FSHD muscle biopsies classified as ‘Mild’ or ‘Moderate’ (Pathology score 0–8), then the mean pathology score classifies both the quadriceps and tibialis anterior in the ‘Mild’ category. In this case, the mean ± SEM proportion of regenerating myofibres in quadriceps was 0.29 ± 0.06% (*n* = 31) and 0.67 ± 0.34% (*n* = 9) for the tibialis anterior, while 32% (8/31) of quadriceps biopsies did not contain a single regenerating muscle fibre.

### Regenerating muscle fibres in all DM2 muscle biopsies

**Figure 5:**
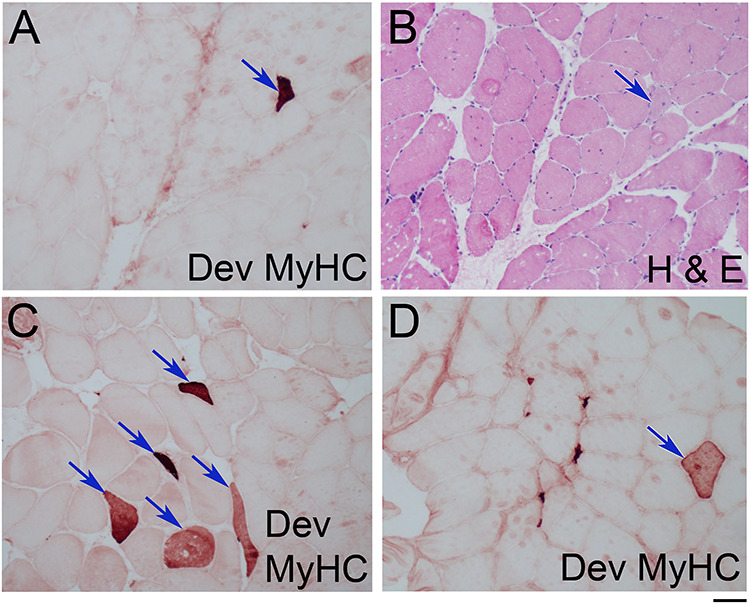
Regenerating muscle fibres in DM2 muscle biopsies. (**A**) Histopathological section from a quadriceps biopsy of a DM2 patient immunolabelled for Novocastra Clone RNMy2/9D2. A regenerating muscle fibre containing Dev MyHC is highlighted by an arrow. (**B**) Adjacent section from the same muscle as in (A), stained with H&E with the regenerating muscle fibre indicated by an arrow. (**C**) and (**D**) Dev MyHC immunolabelled muscle biopsy sections from tibialis anterior muscles from two individuals with DM2, with Dev MyHC-containing regenerating myofibres highlighted by arrows. Scale bars represent approximately 30 μm.

Dev MyHC immuolabelling revealed that four of four DM2 muscle biopsies from quadriceps (Fig. 5A and B) and five of five from tibialis anterior (Fig. 5C and D) contained regenerating muscle fibres (Table 3). There was a mean ± SEM of 1.24 ± 0.41% (*n* = 9) regenerating myofibres per biopsy, with no significant difference between quadriceps and tibialis anterior (*t*-value: 0.027, *P* = 0.98 using logistic regression).

### Muscle regeneration in FSHD correlates with pathology score

The proportion of Dev MyHC containing muscle fibres per patient biopsy did not correlate with patient age or gender, or D4Z4 repeat length in FSHD1, but did correlate with muscle type (Table 4).

Pathologic evaluation on the muscle biopsies also meant that we could determine how muscle regeneration correlated with pathological hallmarks. For DM2, the proportion of Dev MyHC containing muscle fibres did not correlate with either any component of the pathology score or the overall pathology score itself (Table 4).

For FSHD, after adjusting for muscle type (quadriceps or tibialis anterior), the proportion of Dev MyHC containing muscle fibres per needle biopsy was significantly positively correlated with all components of the full pathology score: fibre size variation (*P* = 0.020), central nucleation (*P* = 5.33 × 10^−6^), fibrosis (*P* = 3.04 × 10^−5^) and necrosis/regeneration/inflammation (*P* = 0.007), and hence with the overall pathology score itself (*P* = 5.84 × 10^−6^) (Table 4). There was also an association in FSHD quadriceps between the proportion of regenerating myofibres and the necrosis score (Wilcoxon *P* = 0.032, necrosis 0 versus necrosis 1) but not for the smaller set of FSHD tibialis anterior biopsies.

**Table 1:** FSHD quadriceps muscle biopsies

Subject	Diagnosis	Sex	Age (years)	4qA (kb)	Muscle	Dev MyHC+ per section	Fibres per section	Dev MyHC+ proportion (%)	Assessment of pathology						
									Pathology score (0–12)	Fibre size variation (0–3)	Central nuclei (0–3)	Fibrosis (0–3)	N/R/I (0–3)	Pathology score—(N/R/I) (0–9)	Necrosis (0–1)
20	FSHD1	M	59	32	quad	0	619	0.00	1	1	0	0	0	1	0
31	FSHD1	F	57	18	quad	0	1498	0.00	1	1	0	0	0	1	0
32	FSHD1	M	36	33	quad	2	668	0.30	1	1	0	0	0	1	0
1	FSHD1	M	54	28	quad	3	994	0.30	2	2	0	0	0	2	0
4	FSHD2	F	56	67^*^	quad	2	137	1.46	2	1	0	0	1	1	0
5	FSHD1	F	53	31	quad	3	2178	0.14	2	1	0	0	1	1	0
8	FSHD2	F	69	43^*^	quad	3	412	0.73	2	1	1	0	0	2	0
10	FSHD1	F	29	12	quad	3	801	0.37	2	1	0	1	0	2	0
13	FSHD1	F	60	26	quad	0	2079	0.00	2	2	0	0	0	2	0
14	FSHD1	M	40	29	quad	0	945	0.00	2	2	0	0	0	2	0
17	FSHD1	M	47	25	quad	0	734	0.00	2	2	0	0	0	2	0
18	FSHD1	M	56	23	quad	2	1438	0.14	2	2	0	0	0	2	0
21	FSHD1	F	46	15	quad	6	1725	0.35	2	2	0	0	0	2	0
22	FSHD1	F	47	27	quad	0	1955	0.00	2	2	0	0	0	2	0
7	FSHD1	F	47	35	quad	1	766	0.13	3	3	0	0	0	3	0
19	FSHD1	M	40	28	quad	0	1160	0.00	3	2	0	0	1	2	0
28	FSHD1	M	25	18	quad	0	588	0.00	3	2	0	1	0	3	0
6	FSHD1	M	50	13	quad	1	625	0.16	4	3	0	0	1	3	0
9	FSHD1	F	26	15	quad	1	744	0.13	4	2	1	1	0	4	0
15	FSHD1	F	47	19	quad	1	1672	0.06	4	2	0	1	1	3	1
16	FSHD1	M	63	23	quad	1	1341	0.07	4	3	0	1	0	4	0
27	FSHD1	F	38	28	quad	6	709	0.85	4	1	1	0	2	2	1
34	FSHD1	M	50	34	quad	5	714	0.70	4	2	1	0	1	3	0
3	FSHD1	M	56	17	quad	2	836	0.24	5	3	1	1	0	5	0
11	FSHD1	F	59	10	quad	1	264	0.38	5	3	1	1	0	5	0
23	FSHD2	M	50	46^*^	quad	1	309	0.32	5	2	1	0	2	3	1
26	FSHD1	M	65	27	quad	2	405	0.49	5	3	0	0	2	3	1
24	FSHD1	F	54	24	quad	1	490	0.20	6	2	1	1	2	4	1
25	FSHD1	F	36	25	quad	2	1679	0.12	6	2	1	2	1	5	0
33	FSHD1	F	24	16	quad	12	1300	0.92	6	2	1	1	2	4	1
12	FSHD1 (mos)	M	62	19^**^	quad	3	529	0.57	8	3	1	3	1	7	0
2	FSHD1	M	75	19	quad	13	862	1.51	10	3	1	3	3	7	1
29	FSHD1	M	56	20	quad	5	101	4.95	10	3	3	3	1	9	0
30	FSHD1	M	49	15	quad	3	463	0.65	10	3	2	3	2	8	1
AVG			49.44			2.50	933.53	**0.48**	**3.94**					**3.24**	
SD			12.45			3.06	563.48	**0.88**	**2.55**					**2.05**	
SEM			2.14			0.52	96.64	**0.15**	**0.44**					**0.35**	
*N*			34			34	34	**34**	**34**					**34**	

N/R/I, Necrosis/regeneration/inflammation, SD, standard deviation. *subjects with FSHD2, ** mosaic subjects, bold values in tables 1-3 represent relevant values discussed in text

**Table 2:** FSHD tibialis anterior muscle biopsies

Subject	Diagnosis	Sex	Age (years)	4qA (kb)	Muscle	Dev MyHC+ per section	Fibres per section	Dev MyHC+ proportion (%)	Assessment of pathology						
									Pathology score (0–12)	Fibre size variation (0–3)	Central nuclei (0–3)	Fibrosis (0–3)	N/R/I (0–3)	Pathology score—(N/R/I) (0–9)	Necrosis (0–1)
41	FSHD1	F	53	22	tib ant	2	1173	0.17	1	0	0	0	1	0	1
39	FSHD1	M	64	33	tib ant	0	616	0.00	2	1	0	0	1	1	1
40	FSHD1	F	41	20	tib ant	1	647	0.15	2	2	0	0	0	2	0
35	FSHD1	F	55	29	tib ant	1	428	0.23	3	2	1	0	0	3	0
45	FSHD1	F	66	17	tib ant	1	286	0.35	3	2	1	0	0	3	0
42	FSHD1	M	65	22	tib ant	2	394	0.51	5	3	0	0	2	3	1
44	FSHD1	F	50	19	tib ant	1	1996	0.05	7	3	1	2	1	6	1
38	FSHD2	F	49	43*	tib ant	4	266	1.50	8	3	2	1	2	6	1
43	FSHD1	M	68	27	tib ant	12	388	3.09	8	3	1	1	3	5	0
37	FSHD1	M	38	28	tib ant	25	721	3.47	9	3	2	1	3	6	1
36	FSHD1	M	33	22	tib ant	50	530	9.43	10	3	2	3	2	8	1
AVG			52.91			9.00	676.82	**1.72**	**5.27**					**3.91**	
SD			12.07			15.51	506.10	**2.84**	**3.23**					**2.47**	
SEM			3.64			4.68	152.59	**0.86**	**0.97**					**0.74**	
*N*			11			11	11	**11**	**11**					**11**	

N/R/I, Necrosis/regeneration/inflammation, SD, standard deviation. An asterisk indicates FSHD2. Values in bold text are to highlight proportion of regenerating muscle fibres and pathology scores.

**Table 3:** Myotonic dystrophy type 2 quadriceps or tibialis anterior muscle biopsies

Subject	Diagnosis	Sex	Age (years)	Muscle	Dev MyHC+ per section	Fibres per section	Dev MyHC+ proportion (%)	Assessment of pathology						
								Pathology score (0–12)	Fibre size variation (0–3)	Central nuclei (0–3)	Fibrosis (0–3)	N/R/I (0–3)	Pathology Score—(N/R/I) (0–9)	Necrosis (0–1)
D3	DM2	F	57	tib ant	2	406	0.49	4	2	2	0	0	4	0
D4	DM2	M	61	tib ant	1	678	0.15	5	2	2	1	0	5	0
D5	DM2	F	54	tib ant	1	554	0.18	5	3	1	0	1	4	0
D9	DM2	F	65	quad	11	362	3.04	6	2	2	1	1	5	1
D2	DM2	M	52	tib ant	8	247	3.24	7	3	2	1	1	6	1
D1	DM2	M	62	tib ant	10	452	2.21	8	3	3	1	1	7	0
D6	DM2	M	44	quad	2	580	0.34	8	3	2	3	1	7	0
D8	DM2	F	59	quad	2	243	0.82	8	3	2	1	2	6	0
D7	DM2	F	64	quad	9	1245	0.72	10	3	2	3	2	8	1
AVG			57.56		5.11	529.67	**1.24**	**6.78**					**5.78**	
SD			6.69		4.26	305.85	**1.24**	**1.92**					**1.39**	
SEM			2.23		1.42	101.95	**0.41**	**0.64**					**0.46**	
*N*			9		9	9	**9**	**9**					**9**	

N/R/I, Necrosis/regeneration/inflammation, SD, standard deviation. Values in bold text are to highlight proportion of regenerating muscle fibres and pathology scores.

## Discussion

In this systematic study of muscle regeneration in FSHD, we found that regenerating myofibres were present in 76% of muscle biopsies from quadriceps, with an average of 0.48% regenerating muscle fibres per muscle biopsy for adults. This compares to regeneration being detected in 91% of tibialis anterior biopsies, with a higher mean proportion of 1.72% regenerating myofibres per adult biopsy. Given the caveat of just how representative needle muscle biopsies are of an entire muscle, and since most FSHD biopsies (48/54) were classified as having either mild or moderate pathology, removing those few samples classified as severe may better reflect the general level of regeneration in FSHD. This gave a mean proportion of 0.29% regenerating myofibres in quadriceps biopsies classified as mild/moderate (*n* = 31) and 0.67% for the tibialis anterior (*n* = 9).

The frequency and levels of muscle regeneration in FSHD described here are consistent with the Arahata study, which reported that 67% (12/18) of biceps brachii biopsies showed histological signs of regeneration, with qualitative analysis classifying this regeneration as ‘slight’ in 50% (6/12), ‘mild’ in 33% (4/12) or ‘moderate’ in the remaining 17% (2/12) (31). In a study by Padberg (1) regenerating muscle fibres were found in ~26% (6/23) of biopsies from a collection of either quadriceps, tibialis anterior, triceps brachii, deltoideus and biceps brachii samples obtained from 22 FSHD patients, classifying regeneration as ‘mild’ in all but one case (5/6).

We also examined regeneration at the transcriptomic level using the 200 gene Hallmark_Myogenesis gene set (Supplementary Material, Table S1) generated from 64 studies investigating myogenesis in the Molecular Signatures Database (34). This Myogenesis score was higher in healthy myotubes than in their myoblast counterparts, indicating that it is detecting myogenic differentiation. However, myotubes *in vitro* usually remain immature compared to muscle fibres *in vivo*. They often retain expression of developmental isoforms of proteins such as MyHC, while failing to activate isoforms characteristic of mature myofibres, due in part to lack of innervation/electrical stimulation required for myofibre maturation (53). Thus, the higher Myogenesis score in transcriptomic data from FSHD, DMD and DM2 muscle biopsies is likely due to the presence of myofibres expressing genes characteristic of myogenesis, indicating that they are undergoing regeneration. The Myogenesis score is also a robust means of discriminating dystrophic from control muscle biopsies in all three disorders, with a 76% probability of success in FSHD, 86% for DM2 and 88% for DMD.

Muscle regeneration in FSHD was positively correlated with the overall pathology score, and also with each of its constitutive components of fibre size variation, central nucleation, fibrosis and necrosis/regeneration/inflammation. Muscle regeneration was also associated with the level of necrosis. Serum creatine kinase levels reflect the degree of muscle fibre damage and are usually normal or only slightly raised in FSHD (1). By comparison, DMD generally exhibits high serum creatine kinase levels with severe pathology in many muscles (54). This is accompanied by a robust regenerative response initially in DMD: for example, the proportion of regenerating fibres expressing developmental MyHC isoforms varied from 38 to 47% in quadriceps biopsies from four DMD patients aged 4–13 years (55), 24–33% in muscle biopsies from five DMD patients aged 4.3–8.2 years (20) and a mean of 32% in muscle biopsies from three DMD patients aged 3.3–6.8 years (56). However, DMD clinical onset is within the first few years of life and so these are also growing muscles. Despite this robust regenerative response in DMD though, muscle function is gradually compromised and eventually lost in most muscles.

To gauge if the lower level of regeneration in FSHD compared to DMD is a response to a chronic low-level dystrophic stimulus in an adult muscle, we also examined the similarly slowly progressing DM2. DM2 typically exhibits elevated serum creatine kinase levels. A muscle biopsies from the nine adult DM2 patients that we examined had regenerating myofibres. The mean proportion of 1.24% regenerating muscle fibres per biopsy was not significantly different to FSHD. Unlike FSHD, however, the proportion of regenerating muscle fibres in DM2 was not correlated with the overall pathology score, or individually with any of its four constituent measures, although there were fewer samples than for FSHD.

FSHD and DM2 are slowly progressing, yet muscle regeneration is insufficient to maintain muscle bulk and function in certain muscles. As discussed, this could be a result of a low level of stimulus to satellite cells, combined with an increasing hostile microenvironment (26). However, it could also be that the regenerative abilities of satellite cells are also directly compromised in these disorders. Satellite cells are present in FSHD quadriceps (specifically vastus lateralis) biopsies (*n* = 10) with a median 0.19 satellite cells per myofibre, not significantly different from the 10 control biopsies (29). However, primary FSHD myoblasts often exhibit perturbed myogenesis *ex vivo*, forming myotubes classified as either ‘atrophic’ or ‘disorganized’ (57). Our image analysis of myogenic differentiation in FSHD shows a failure to reach the size of healthy myotubes, revealing that the myotube phenotype is more hypotrophic (37). The Myogenesis score is lower in confluent immortalized FSHD myoblasts compared to controls, which may reflect a delayed entry into myogenic differentiation. Consistent with this, an early microarray-based transcriptomic study found that many genes engaged in differentiation were altered in FSHD muscle, suggesting a partial block in the myogenic differentiation program (58). Moreover, a recent RNA-seq-based transcriptomic study identified a subset of FSHD samples displaying a ‘muscle-low’ transcriptome, suppressing genes typically involved in myogenesis (59). For DM2, myoblasts from adult DM2 patients differentiate as effectively as controls *ex vivo* (60–62) but are characterized by a premature proliferative growth arrest, halting cell division earlier than controls (61).

**Table 4:** Correlations to proportion of Dev MyHC+ muscle fibres

Correlations of data to proportion of Dev MyHC+ myofibres		
Continuous variables	Pearson’s *r*	*P* value
Age	−0.044	0.749
D4Z4 (FSHD1 only)	−0.017	0.920
Binary variables	*t*-score	*P* value
Gender	1.686	0.098
FSHD versus DM2	−0.779	0.440
Muscle type	2.239	0.029
FSHD: Correlations (adjusted for muscle type) to proportion of Dev MyHC+ myofibres		
Continuous variables	Pearson’s *r*	*P* value
Fibre size variability	0.365	0.020
Central nucleation	0.651	5.34E−06
Fibrosis	0.609	3.04E−05
Necrosis/regeneration/inflammation	0.420	0.007
Pathology score	0.649	5.84E−06
DM2: Correlations (adjusted for muscle type) to proportion of Dev MyHC+ myofibres		
Continuous variables	Pearson’s *r*	*P* value
Fibre size variability	−0.077	0.793
Central nucleation	0.089	0.762
Fibrosis	0.089	0.763
Necrosis/regeneration/inflammation	0.225	0.439
Pathology score	0.124	0.673

Significant correlations (*p* < 0.05) are highlightedt

FSHD1 and FSHD2 manifest as a coherent clinical condition through ectopic expression of DUX4 (7,8,10), which could also affect the regenerative response to dystrophy since DUX4 is toxic to many cell types across numerous species (63,64). Two myogenic enhancers proximal to D4Z4 (65) likely explain *DUX4* expression in skeletal muscle, hence FSHD manifesting as a muscular dystrophy (7). DUX4 can also be detected, albeit at very low levels, in myogenic cells from FSHD patients (17,66,67). Primary FSHD human myoblasts express *DUX4* and DUX4 target genes, with levels then increasing as myoblasts differentiate into myocytes and myotubes (68,69). Expression of *DUX4* also increases during acute muscle regeneration in the D4Z4-2.5 mouse model (70), transgenic for a contracted human 2.5 D4Z4 unit region obtained from an FSHD-affected individual (71). The effects of DUX4 on myoblasts include inducing a stem cell-like transcriptome, perturbing/inhibiting myogenesis and causing differentiation into hypotrophic myotubes (66,70,72).

FSHD muscle is characterized by a progressive suppression of PAX7 transcriptional target genes (38,73,74), an important observation as PAX7 is a master regulator of satellite cells (75). The homeodomains of DUX4 show homology with the homeodomain of PAX7, and a competitive interaction has been shown between DUX4 and PAX7 proteins (72,76). The PAX3 or PAX7 homeodomain can also substitute those of DUX4 without affecting certain functions of DUX4 (76). As DUX4 interferes with the function of PAX7 and the closely related PAX3 (38,72), it is likely that such interactions during development and/or in satellite cell-derived myoblasts may reduce the effectiveness of regeneration. Thus, it is likely that muscle damage in FSHD is ultimately caused by ectopic DUX4 and that such damage elicits a proportional satellite cell-mediated repair response. However, proliferating and differentiating satellite cell-derived myoblasts may then also be compromised by DUX4 expression/suppression of PAX7 target genes, in addition to operating in an increasingly hostile dystrophic microenvironment manifesting chronic inflammation and fibrosis.

There are several potential therapies in development and/or clinical trial to suppress DUX4 expression in FSHD (77). Therapeutic reduction of DUX4 will hopefully slow/prevent muscle fibre damage to suppress further muscle weakness and wasting. Suppression of DUX4, though, may also release regenerative potential. Knowing that there is a regeneration response in many FSHD patients means that regenerative therapies could also be employed to further support restoration of muscle function (37).

In summary, we report that FSHD, DM2 and DMD muscle is characterized by a transcriptomic signature demonstrating ongoing myogenesis, indicative of active muscle regeneration. The majority of muscle biopsies from FSHD patients contain developmental MyHC-containing regenerating muscle fibres, the proportion of which correlates with the severity of pathology.

## Material and Methods

### Gene expression data sets

The Broad Institute gene set called HALLMARK_MYOGENESIS (78) was downloaded from http://software.broadinstitute.org/gsea/msigdb/cards/HALLMARK_MYOGENESIS.html (Supplementary Material, Table S1). Transcriptomic data from muscle biopsies were obtained from the GEO database (https://www.ncbi.nlm.nih.gov/geo/) and included four DMD [GSE1004 (41), GSE3307 (42), GSE38417, GSE6011 (43)], seven FSHD [GSE36398 (44), GSE3307 (42), GSE26852 (45), GSE10760 (46), GSE9397 (17), GSE56787 (47), GSE115650 (48)] and five DM2 [GSE7014 and GSE13608 (49), GSE37794 (50), GSE45331 (51), GSE47968 (52)] data sets. Transcriptomic data from FSHD and control myoblasts and myotubes were from GSE123468 (37), GSE102812 (38) and GSE153523 (39).

### Meta-analysis of target gene scores

Normalized data for all publicly available microarray and RNA-seq studies were obtained from the GEO database. Quantile log-normalization was subsequently performed on data from each study separately. To enable the evaluation of the Myogenesis score, probes in each microarray data set and sequences in the RNA-seq data were matched to unique EntrezGene identifiers. Probes or sequences mapping to the same gene identifier were averaged.

For single studies, a two-tailedWilcoxon U-test was performed to assess significance. Meta-analysis to assess the discriminatory power of the Myogenesis score was performed across gene expression data sets from the three muscular dystrophies and their control muscle biopsies. *P* values denoting the significance of the scores on meta-analysis were derived from a Fisher’s combined test. ROC curve analysis was performed using the pROC package in R (79).

### FSHD and DM2 muscle biopsies

The FSHD study cohort included 45 subjects (49% female) with genetically confirmed FSHD1 (*n* = 41) or FSHD2 (*n* = 4). The mean subject age was 50 years (range: 24–75 years) (Tables 1 and 2). The genetically confirmed DM2 study cohort included nine subjects (56% female) with a mean subject age of 58 years (range: 44–65 years) (Table 3).

All study subjects consented to needle muscle biopsy procedures under a study protocol approved by the Institutional Review Board of the University of Rochester Medical Center, NY, USA. Needle muscle biopsies were obtained from either the tibialis anterior (*n* = 11) or quadriceps (*n* = 34) muscles from FSHD1 or FSHD2 patients and from tibialis anterior (*n* = 5) or quadriceps (*n* = 4) from the nine DM2 patients, comprising an average ± SEM of 814 ± 73 muscle fibres per biopsy.

### Grading of muscle biopsy pathology

The 10 μm cryosections from the muscle needle biopsies were stained with H&E or trichrome and graded for pathologic changes. A pathologic severity score was assigned based on a 12-point scale giving a 0–3 score to each of four histologic features: variability in muscle fibre size, proportion of centrally located nuclei, interstitial fibrosis and necrosis/regeneration/inflammation (36). FSHD samples were also separately scored for necrosis.

### Immunolabelling

Cryosections of the FSHD and DM2 muscle biopsies were immunolabelled using Novocastra NCL-MHCd (Clone RNMy2/9D2), which recognizes a MyHC present during embryonic and neonatal periods in skeletal muscle development and transiently during myofibre regeneration. On each slide of multiple FSHD or DM2 cryosections, we also included a positive antibody control (muscle biopsy from a patient with a necrotizing myopathy that has many regenerating muscle fibres) and negative antibody controls (muscle biopsies from two healthy individuals). Novocastra NCL-MHCd was applied at 1:10 for 60 min at room temperature and washed 2 × 1 min in Tris Buffer before incubating with 4 plus Biotinylated Universal Goat Link (Biocare Medical cat # HP504US) for 20 min at room temperature. Cryosections were then washed 2 × 1 min in Tris Buffer, followed by 4 plus Streptavidin HRP Label (Biocare Medical cat # HP504US) for 10 min at room temperature. After 2 × 1 min Tris Buffer washes, cryosections were incubated in ImmPACT® NovaRED™ Peroxidase (HRP) Substrate (Vector Laboratories cat # SK-4805) for 10 min at room temperature. Then washed 2 × 1 min in Tris Buffer and 2 × 1 min in water, before dehydration in 95 and 100% alcohol, clearing in Xylene substitute for 5 min and mounting using Fisher Chemical™ Permount™ (Thermo Fisher Scientific cat # SP15–100).

A regenerating myofibre was identified by a brown precipitate, indicating immunolabelling for Dev MyHC. As the sarcoplasm of necrotic fibres can non-specifically bind antibodies, Dev MyHC myofibres were also examined on an adjacent H&E-stained section and excluded if necrotic. The number of muscle fibres per biopsy was determined using the H&E staining to permit the number of regenerating fibres to be expressed as a proportion. For DM2, since immunolabelled and H&E sections were simultaneously examined to confirm a muscle fibre as regenerating, it was not possible to blind evaluation due to the characteristic pathology of DM2.

### Statistical analysis

Correlation analysis between the proportion of Dev MyHC containing myofibres and continuous variables (age, D4Z4 repeat length, pathology score and components) was performed using Pearson correlation. For binary variables (gender, muscle type, disease status), we employed logistic regression. To determine associations between the proportion of Dev MyHC containing myofibres and the pathology score and its components independently of muscle type, the residuals of a logistic regression associating proportion of Dev MyHC containing myofibres with muscle type were employed in Pearson correlations in place of the unadjusted proportion of Dev MyHC+ myofibres. All analysis was performed in R.

*Conflict of Interest statement*. The authors have declared that no conflict of interest exists.

## Supplementary Material

Banerji_et_al_2020_Supplementary_Table_S1_Hallmark_Myogenesis_gene_list_ddaa164
